# “Embracing the present and fearing the future”: The meaning of being an oldest old woman in a rural area

**DOI:** 10.3402/qhw.v9.25217

**Published:** 2014-10-29

**Authors:** Tove M. Ness, Ove Hellzen, Ingela Enmarker

**Affiliations:** 1 Department of Nursing, Mid-Sweden University, Østersund, Sweden; 2 Department of Health Sciences, Nord-Trøndelag University College, Namsos, Norway; 3 Centre of Care Research, Steinkjer, Mid-Norway

**Keywords:** Home nursing care, oldest old, phenomenological hermeneutics, rural

## Abstract

In Western countries, the number of older people receiving home nursing care is increasing, and in rural areas they are at additional risk because of the distance between people and health care facilities. The aim of this study was therefore to illuminate the meaning of being an oldest old woman living alone in a rural area and receiving home nursing care. A sample of 11 oldest old women living in rural areas in the middle of Norway was chosen for this study. Narrative interviews were conducted, and the data were analyzed using the phenomenological hermeneutic method. After a naïve reading and a structural analysis of the text, we identified four themes: *being satisfied with life, being thankful, feeling vulnerable*, and *feeling secure*. The comprehensive understanding implied that being an oldest old woman living alone in a rural area meant living in the intersection between embracing the present in solitude and fearing the future with additional declining health. Living in this complex situation meant to enjoy the present, but still fear the future, as the oldest old women knew their present life situations were limited. This challenging emotional situation meant using their inner strength by trying to be optimistic and seeing opportunities in present life, even if losses were many and extensive. By using their inner strength in facing losses and declining health, the oldest old women managed to appreciate aloneness as solitude, and find new meaning in life.

Getting older reflects a wide range of biological, psychological, and social changes in life (Johnson, Bengtson, Coleman, & Kirkwood, 2005). Next to declining health and increasing age, loss of one's spouse, close friends, and family members becomes a major issue. Above all, losses can imply social isolation and therefore a big challenge to several older people. Social isolation refers to objective physical separation from other people, such as living alone or residing in a rural geographic area. Due to this, older people living alone are seen as a vulnerable group in terms of residential and social isolation, and they face different challenges (e.g., more difficulties with carrying out daily activities because of the lack of benefits from sharing life with a co-resident) (Gaymu & Springer, 2010).

Worldwide, there is a rapidly increasing population of oldest old people (80 years and above), and, despite declining health, there has been a significant increase in older people living alone in their own homes in recent decades (Chandler, Williams, Maconachie, Collett, & Dodgeon, 2004). For example, in Norway, six out of 10 persons older than 80 years live alone (Andreassen, 2011).

Home is often theorized as multidimensional and defined as “a dwelling place or a lived space of interaction between people, places and things; or perhaps both” (Mallett, 2004, p. 84), and a place in which one can be oneself, feel safe, and create one's routines (Silfverberg & Ternestedt, 2007). Home is value laden (Mallett, 2004) and of great importance to older people (Oswald & Wahl, 2005). The home environment holds a central place in the lives of older people (Dahlin-Ivanoff, Haak, Fänge, & Iwarsson, 2007), and it provides a context for many valued roles and activities (Haak, Dahlin-Ivanoff, Fänge, Sixsmith, & Iwarsson, 2007a), as older people value a home that supports their doing and being (de Jonge, Jones, Phillips, & Chung, 2011).

Russell (2007) stresses that home has a different meaning to older men and older women. Historically based gender roles for today's cohort of oldest old people were that the women stayed at home and men went out to work (Oswald & Wahl, 2005). This is also the case for today's cohort of oldest old women in rural areas in Norway. The older women were raised with clear expectations of their gender-based roles as wives and mothers, and many dedicated themselves to the “making of a home” and raising their family (Shenk, Kuwahara, & Zablotsky, 2004), and they invested enormous amounts of emotion and time in “the making of a home” (Howell, 1994). This has resulted in the women being more locally bound to the home than men (Russell, 2007) and more familiar with the territory by everyday behavior (Oswald & Wahl, 2005). Because of this, the importance of home may be more salient to older women's self-identity (Leith, 2006).

In Western countries, the number of older people receiving home nursing care is increasing, and the number of older people living at institutions is decreasing (Tarricone & Tsours, 2008). Older people living alone are also more likely to get help from home nursing care, friends, and family (Otnes, 2011). In Norway, home nursing care means help with, for example, personal hygiene, medication, wound care, and meals (Johansen & Fagerström, 2010).

In rural areas, they are at additional risk because of the distance between people and health care facilities, which can result in geographic isolation. Despite this, studies show that oldest old rural adults living alone define themselves and their health in terms of their homes, and they emphasize that home is where their health is, which also underlines the value of independence (Hayes, 2006). Living alone can give rise to feelings of both loneliness and solitude. A life in solitude means seeing the positive aspects of aging and loss and experience satisfaction with life (Powys, 1974). Hinck (2004) also stresses that oldest old rural adults define their health by their ability to function, rather than recognition of pain and illnesses.

Ness, Hellzen, and Enmarker (2014) found that being an oldest old man living alone and receiving home nursing care in rural areas mean living in a struggle between a dependent existence and a desire to be independent. The wish to not be a burden and maintain independence is important to older people (e.g., Sundin, Bruce, & Barremo, 2010). For example, oldest old men search outside their home for significant others after the loss of their spouse in order to, for example, fight loneliness (Ness et al., 2014). Most of these men had relatives in the same community, even if closeness to relatives varied in distance and degree of contact.

Illuminating how oldest old women living alone in rural areas and receiving home nursing care, who are additionally physical isolated because of physical distance from relatives, therefore became of importance. More women than men reach the age group of the oldest old, yet no specific studies about this theme have been found. Therefore, the aim of this study was to illuminate the meaning of being an oldest old women living alone in a rural area and receiving home nursing care.

## Method

A qualitative approach was chosen for this study because, when studying peoples’ experiences and seeking to understand their lives and worlds, we have to ask them to tell us, so that we can be allowed to hear their perspective and seek to understand their point of view (Patton, 2002). The aim is not to generalize, but to see the uniqueness in everybody's situation. This method applies an inductive style, in which participant perspective forms the basis for new knowledge (Creswell, 2009).

### Participants

A sample of 11 oldest old women from 82 to 95 years of age and living in the rural areas in the middle of Norway was chosen for this study. There is no unambiguous definition of rural (Berg & Lysgård, 2004), but in this study rural is defined as fewer than five inhabitants per square kilometer and a travel time of more than 45 min to a density populated area of over 3000 people. The informants lived in six different municipalities. Occupational backgrounds varied from housewives to smallholders. Closeness to relatives varied, but nobody saw their relatives on a daily basis because of distance. All of the women had lost their spouse, some in recent years, and others several decades ago. Almost all deceased spouses had worked outside the home, while the informants were busy in “the making of a home.” All of the women lived alone and received home nursing care. Some had visits from home nursing care several times a day, others once a week.

To gain access to the informants, the first author contacted the health care leader in each municipality, who then communicated the information to the home nursing office. The home nursing care staff then contacted possible informants and informed them about the study, and arranged contact with those who were interested in participating. After that, the first author arranged the meeting, but the informants decided the time and place. The informants were informed verbally and by written information about the purpose of the study, both by home nursing care staff and by the first author.

### Interviews

The first author conducted narrative interviews (Mishler, 1986) from spring to autumn 2013, asking the older women to narrate stories regarding specific themes:

- Please, can you tell me about your experience of receiving home nursing care?
- Please, can you tell me about your experience being an old woman today?
- Please, can you tell me about how you experience living alone?

When trying to understand other people's perspectives on life, listening to their stories is crucial, because we are the stories we tell (Sandelowski, 1994). Telling their story also encourages people to speak in their own “voice” (Mishler, 1986). To illuminate the meaning of being an oldest old woman, narrative interviews could contribute to gathering deeper descriptions about the research phenomenon. During the interviews, the interviewer asked clarifying questions only when she did not understand or when deeper reflections were needed. The interviews were carried out in the informants’ own homes and lasted 93–160 min. The interviews were recorded and transcribed verbatim by the first author.

### Data analysis

To reveal the meaning of being an oldest old woman, the phenomenological hermeneutic method developed by Lindseth and Nordberg (2004) and inspired by Ricoeur (1976) was used. Ricoeur (1976) emphasizes that, through interpretation, we have to understand the author better than he understood himself. We have to find the meaning in front of the text.

Firstly, a naïve reading is done to grasp a naïve understanding, or a guess, as Ricoeur (1976) argues. This can be one of several possible interpretations. In this phase, the transcribed interview text was openly and separately read several times in order to grasp the text's meaning as a whole. This reading offered a first naïve understanding of each interview text—a naïve understanding of the meaning of the lived experiences of being an oldest old woman, living alone in a rural area, and receiving home nursing care.

The second step, the structural analysis, aims to validate or invalidate the naïve understanding, and subthemes and themes are then formulated (Lindseth & Nordberg, 2004). Here, the interpretation continued in order to validate or invalidate the naïve understanding and identify and formulate subthemes. The thematic analysis began with each interview text, and was worked through sentence by sentence. Meaning units in accordance with the study aim and the naïve understanding were identified and divided. Each meaning unit was then condensed into everyday language and abstracted. For structures to emerge, each meaning unit was reflected upon, and then divided into subthemes and themes. An example is given in Table I.

**Table I:** Example of the data analysis process

Meaning unit	Condensed meaning unit	Subtheme	Theme
I use time on memories, and then especially about my deceased spouse.	Use time on deceased spouse	Finding meaning	Being satisfied with life
I enjoy being alone. I don't need others to feel good. I have had much to do, and did much needlework earlier, but not so much now.	Enjoy aloneness. Don't need others. Much to do. Needlework earlier.	Appreciating aloneness	Being satisfied with life
I don't think much about dying, but I hope I don't get much pain in the end. My husband had a lot of pain, and he suffered a lot.	Don't think about death, but hope to not get much pain. Husband pain.	Afraid of future	Feeling vulnerable

The last step is to develop a critical interpretation so one can formulate a comprehensive understanding and therefore interpret the text as a whole (Lindseth & Nordberg, 2004). Here, the naïve understanding and the structural analysis of being an oldest old woman living alone in a rural area and receiving home nursing care were joined together in order to reach a comprehensive understanding of the whole. In order to reach a comprehensive understanding, the authors’ pre-understanding, the research question, the context of the study, and relevant literature were applied.

### Ethical considerations

The informants were thoroughly briefed beforehand and guaranteed confidentiality, and no individual characteristics are disclosed in reporting the results. The data were stored in a locked facility to which only the first author had access. Each informant gave informed consent to participate before she participated in the study. Permission for research was granted by the Norwegian Social Science Data Services (No. 28728) and carried out in accordance with the Declaration of Helsinki (World Medical Association, 2008).

## Findings

### Naïve reading

The oldest old women are strongly attached to their home, so despite diseases and impairments, home is where their health is. This means that home is where they feel safe and content. Home is where their aloneness leads to qualitative time in solitude, with reading, flowers, needlework, resting, television, and cleaning, for example. This time in solitude is considered meaningful for the oldest old women. So, even if they appreciate spending time with others, being at home is more important to them.

The oldest old women are focusing on today, but they also have some fears about the future. They try to find practical solutions to the challenges that diseases and impairments bring to everyday life. This means that they are focusing on what they can do and the positive aspects of aging. The help they receive from home nursing care is considered valuable, because it means they can stay longer in their own homes, and do not have to move to a sheltered housing or the local nursing home.

### Structural analysis

The results of the structural analysis are shown in four themes and 11 subthemes. The themes summarize the oldest old women's views of being alone in their homes and receiving home nursing care.

### Theme: Being satisfied with life

This theme reflects the oldest old women's satisfaction with their present life, despite losses, declining health, increasing age, and short remaining life span. So, despite a challenging everyday life, the women accept, appreciate, handle, and find meaning in their life as an oldest old woman.

*Accepting their present life situation means that oldest old women accept their current life situation* in aloneness with diseases and impairments. One of the consequences of being an oldest old woman is to experience losses, for example the loss of spouse, siblings, and friends, to death or disease such as dementia or stroke. The women have accepted and feel comfortable in their aloneness. Accepting their present life situation in aloneness also means accepting that relatives cannot come around so much because of their distance from the oldest old women. Being alone is preferable to moving to sheltered housing or a nursing home to be with others, and they do not feel lonely.

I don't feel lonely. One must take life as it comes. If you become alone, then you become alone. That's how it is.

Oldest old women also see and accept diseases and impairments as a part of becoming an oldest old woman. This means they feel that it is natural to get diseases and that their body is worn out from earlier physical labor and it is something all experience with age. Condoning their present life situation with disease and impairment means reconciliation with the fact that they cannot participate or perform as before because of decreasing health. For example, walking in the woods, doing different chores in the house, or grooming themselves as they used to are more difficult than in earlier years.

*Handling and seeing opportunities in one's own life* mean handling and seeing opportunities in one's own life as an oldest old woman despite diseases and impairments, by focusing on their resources and what they could still manage. For example, by using different aids, oldest old women could still do parts of chores (e.g., different housework or personal hygiene). Handling and seeing opportunities in one's own life mean being adaptable to changes that a new impairment or disease can represent, and not giving up but trying to see opportunities despite decreasing health. By trying to see changes as a possibility in growing older, they are handling their life situation as oldest old women with decreasing health.

I always had to reconsider when something has happened to me, but that's how it is, but still it's important to do the things you can do.

By considering one's health condition the actual day before deciding what to do, the oldest old women were handling their diseases and impairments (e.g., by postponing or pushing forward a chore). This means being patient and resting on the actual day if necessary. Having an optimistic outlook on life, despite the limitations that declining health can represent, oldest old women are able to see opportunities in their present life situation even if losses can be many and extensive.

*Appreciating aloneness* means feeling comfortable in one's one company, and not feeling lonely but appreciating time alone. Alone oldest old women, for example, read, do parts of different chores, and relax. Appreciating aloneness means they do not need the company of others to feel content in everyday life as an oldest old woman. Appreciating aloneness also means preferring and enjoying being alone even when they have opportunities to be with others (e.g., by sitting in their comfortable chair and feeling relaxed).

I enjoy being alone. It's good to think that I don't have to be elsewhere.

Finding positive aspects of aloneness entails feelings of appreciation for solitude rather than feelings of loneliness. Contact with children and friends on the telephone counteracts feelings of loneliness in their aloneness as well as, for example, closeness to the nearest road.

*Finding meaning* means finding meaning in everyday life alone, when declining health and impairment have made them more homebound than in earlier years. Now, as oldest old women, they find meaning in different chores, for example doing parts of housework, with some help from the home helper or the home nursing care.

I cook dinner, and different other chores in the house, and some needle working.

Earlier, many of the women had to sew clothes and knit to make ends meet, but now their skills help them to find meaning in life as an oldest old. Yesterday's chores have become today's hobbies. Declining health has made them look for meaning in other hobbies or parts of chores than before, and most women see possibilities when seeking something to fill their time with. Different aids make this easier, such as using audiobooks or a magnifying glass. Oldest old women also find meaning and pleasure in leaning on their earlier memories (e.g., of their deceased spouse). God has also given rise to comfort and meaning when life has been challenging and demanding for the oldest old women, as everything happens for a reason.

### Theme: Being thankful

This theme reflects the oldest old women's thankfulness toward their present life as an oldest old woman and their gratitude toward home nursing care for received care.

*Gratitude toward life* means their thankfulness toward life for their perceived good health despite impairment, diseases, and chronic pain. The oldest old women are satisfied with their life from early childhood in spite of different adversities. Now, as oldest old women, they feel gratitude toward life for being able to stay in their homes and receive a retirement pension. Gratitude toward life is also shown in their thankfulness over being able to do parts of different chores (e.g., washing clothes or taking bedclothes off the bed). Having the ability to fend for themselves (e.g., getting into and out of bed) gives them great pleasure and feelings of coping and gratitude toward life. Access to different aids enables this gratitude. Oldest old women also feel gratitude and consider themselves lucky for not having more severe diseases such as dementia.

My health is good, but my feet are not as they used to be, so I can't go so fast, but that's nothing to worry about. My head is good though.

*Appreciating support* means their appreciation toward home nursing care for the practical and psychological care given to them in everyday life, which enables the oldest old women to stay in their beloved homes.

It's ok to be old and live alone when you get some help …//… I do not need anything else.

The oldest old women's appreciation of support also means their gratefulness for all of the different characteristics that the formal caregivers have (e.g., kind, supportive, skilled, polite, helpful, reassuring, and humorous). This means that all caregivers in home nursing care are important and considered valuable to them, and they do not prefer one over the other, but appreciate all with their different qualities.

### Theme: Feeling vulnerable

This theme reflects the oldest old women's attempts to live for the moment as they try to displace their fear for tomorrow with increasing feelings of bodily decline.

*Living for the moment* means trying to take care of the moment because they know time as an oldest old woman is limited. Decreasing health and increasing age mean they have no promise of tomorrow. Living for the moment means enjoying the present moment, taking one day at a time, and not taking sorrows in advance.

There will be some losses in a life, and you don't know when, you must take the days as they come. Don't mourn about tomorrow, because every day has enough with its own sorrow.

Losing different bodily functions is something the oldest old women try not to think about, as they try to live for the moment. Living for the moment also means appreciating and getting the most out of every day, while still trying to be prepared for the changes that age and decreasing health bring. Because they know time is limited, living for the moment is essential for the oldest old women.

*Suffering because of diseases and impairments* means feeling the consequences of diseases and impairments in everyday life as an oldest old woman. Having diseases and impairments affects the oldest old women to varying degrees, but it contains losses because they have to renounce things that earlier gave them great pleasure.

I liked to dance before, but now my feet will not follow me …//… My stroke has affected my ability to speak as well.

Oldest old women are restricted by diseases and impairments, and daily chores and mobility become challenging. Things that were easy to do before become challenging tasks, because of the consequences that the diseases and impairments bring in everyday life.

*Afraid of the future* means the oldest old women's fear of having to move from their beloved homes, as their health decreases and new diseases and impairments arise. Every new impairment or disease represents a fear of having to move from their home. One dominant fear is the fear of becoming demented.

It's most important that my head works, not the feet. When the head don't work anymore, then there is nothing more.

Being an oldest old women means that time left is limited, and this is something the oldest old women acknowledge. Even if they don't fear death, they fear the period prior to death, especially developing severe pain. Being an oldest old, all of these women will follow their spouse and other peers to their grave. They have seen or heard about challenging times at the end, and they do not want the same for themselves.

### Theme: Feeling secure

This theme illustrates the oldest old women's feelings of security when being in their own homes, and having home nursing care as backup when needed.

*Importance of home* contains the oldest old women's strong connection to their homes. This means, for example, feelings there will always be a solution if they get home from the hospital or the nursing home after a disease event. Here is where they find strength and peace. Importance of home means having a place to retreat and always feel welcome and safe.

My home means everything to me. When I get there, it's always a solution.

The oldest old women have been in their homes since early adulthood, some from childhood. Now, as oldest old women, they feel they belong to the houses and the place, even if several places and buildings have decayed with time. Here, they can lean on memories of their children and former spouse, and this is essential for several women.

*Having support* means having home nursing care as backup when something happens. Oldest old women do not have to consider who or when to call when support is needed, because they have the opportunity to call home nursing care for practical and psychological support, day and night. Having the home nursing care for support means that oldest old women have someone skilled with whom to discuss their health condition or need for practical or psychological help, and that means a lot to the women.

They mean everything to me. I know they come, and if it's something, I can talk with them about it.

Having support means that the oldest old women can stay in their homes because of practical and psychological support from home nursing care. This means that they feel confident they can get the support they need when requested.

### Comprehensive understanding

Being an oldest old woman living alone in rural areas means living in the intersection between embracing the present in solitude and fearing the future with further declining health. By using their inner strength, they are turning aloneness into positive time in solitude, rather than negative time in loneliness. The oldest old women are enjoying aloneness by finding meaning in everyday life, with, for example, needlework and different chores. By finding meaning in their aloneness, the oldest old women manage to take pleasure in the present despite the restrictions that age, losses, diseases, and impairments represent. However, declining health and increasing age mean limited time left in their beloved homes, and this is considered a major challenge for the oldest old women, despite support from home nursing care. Being an oldest old woman means knowing time left in their homes is limited, so the present is considered valuable. Living in the intersection between embracing the present and fearing the future means living in a challenging emotional situation, being thankful and satisfied, feeling secure, and yet feeling vulnerable.

## Discussion

Despite adversities and fears about the future with further declining health, the oldest old women manage to find meaning and *are satisfied with life*. A positive life orientation is a decisive inner resource for the health and well-being of older people, including the ability to find meaning in life and look forward to and have plans for the future (Fagerström, 2010), even if older people have a tendency to appreciate small things in daily life (Andersson, Hallberg, & Edberg, 2008). In spite of age and decreasing health with reduced capacity and functional constraints, being occupied and staying healthy and of sound mind are considered important to manage most independently in the doings of everyday life as an oldest old person (Larsson, Haglund, & Hagberg, 2009).

Several studies have found a clear connection between satisfaction in life as an old person and a strong feeling of resilience. Windle (2011) argues that resilience is the process of adapting to, negotiating, or managing significant sources of trauma or stress. The individual's assets, resources, life, and environment therefore become of great importance, as these enable their ability to “bounce back” in the face of adversity.

Oldest old people with estimated high resilience are able to feel connected and have positive feelings, satisfaction, and gratitude in growing old. They also feel more independent, being able to ignore impaired bodily experiences and instead focus on cognitive abilities. They are still able to create meaning in their life as oldest old by doing handicraft and housework, and living in relation to memories (Alex, 2010). This is congruent with our findings; despite an impaired body that makes them more homebound, the oldest old women are able to find meaning in their aloneness. Oldest old men in rural areas also find new meanings in life after loss of their spouse, for example by searching outside their home for significant others at the local nursing home or at the local café (Ness et al., 2014).

Sense of coherence is a dimension of resilience that can be seen in people facing adversities throughout the lifespan (Windle, 2011), and Söderhamn, Dale, and Söderhamn (2011) found that older people who live in rural areas and have a strong sense of coherence experience adversity (e.g., loss of spouse or a child) with a sense that life must go on. Accepting the variety of days and not feeling sorry for oneself are important factors in feeling well as an older person living alone in rural areas (Söderhamn et al., 2011). This is congruent with our study, as the oldest old women accept their current life situations, for example not being able to see their children so often because of distance. So, being satisfied, having a positive outlook on life, and being happy with one's situation (i.e., being an optimist) are important factors for having a good life and feelings of well-being as an old person (Söderhamn et al., 2011).

Oldest old people's ability to adapt to and find new meaning in life and in new conditions such as declining health, emerging dependency, and loss is significant for the feeling of anchorage to live (Borglin, Edberg, & Hallberg, 2005). Findings in our study showed that, despite decreasing health and increased impairments, the oldest old women were able to be optimistic and satisfied with life, and to focus on opportunities rather than limitations in present life situations. Having meaning in life and equanimity may be an advantage for oldest old people as they meet the challenging situations of illness and growing older (Moe, Ekker, & Enmarker, 2013a).

Even if receiving care causes mixed feelings for those who receive it, receiving home nursing care gives rise to being thankful for the majority of the oldest old (e.g., Dale, Sævareide, Kirkevold, & Söderhamn, 2011; Moe, Hellzen, & Enmarker, 2013b). In our study, the oldest old women were *being thankful*, and this was especially shown in their appreciation for having support from the home nursing care, even if Moe et al. (2013b) stresses that receiving home nursing care is a field of emotional contradiction, sometimes promoting dignity and at other times diminishing it.

Being thankful was also demonstrated in their gratitude toward life, as older people often feel gratitude for having the opportunity to get up every day and feel well (Dale, Söderhamn, & Söderhamn, 2012). This is congruent with our study, as the oldest old women were grateful toward life, and they were grateful for such things as having the ability to get up in the morning and not having dementia.

The oldest old women were *feeling vulnerable* because of fears about the future and declining health, especially the fear of becoming demented and having to move from their homes. This concurs with Dale et al. (2012), who found that older people living alone in rural areas in southern Norway were particularly concerned about the possibility of becoming cognitively impaired, and then losing the ability to remain in their own homes.

As the oldest old women knew they have limited time left, they felt vulnerable, as they were trying to live for the moment and not take sorrows in advance. Living for the moment and in the present is a coping strategy for preserving health and continuity in the lives of old women, and it is a strategy for overcoming the uncertainty of the future that is associated with aging (Hayes, 2006). The oldest old persons in their eighth and ninth decades recognize and accept an uncertain future, as they live for the present, knowing lifetime is limited (Hinck, 2007).

Borglin et al. (2005) found that living for the present meant taking each day as it comes and not making plans for the future. Cultivating a positive outlook offered a sense of meaning among the oldest old people. This was congruent to our findings, but the women were suffering because of the effects of diseases and impairments on their everyday lives as oldest old people. That is, they had to renounce things that had formerly given them great pleasure. They had to find new meaning in life. Frankl (1971) stresses that man in aloneness has the ability to create a new world by connecting the past and future and imbuing it with new meaning after adverse experiences in life, which loss of one's spouse and declining health can represent. Our attitude in adverse life situations can help us perceive meaning and purpose and reduce despair in challenging circumstances. We can, as Frankl (1971) expresses it, choose how to react in each situation in life.

Older women in rural areas struggle to keep their freedom, and in their struggle, home can be a buffer for many. To lose one's home is to lose one's self (Gattuso, 1996). Our study revealed oldest old women's feelings of vulnerability, which was seen in their fears for the future and their attempt to live for the moment, despite suffering because of decreasing health. This means that their homes were essential to the women, and meant independence and valuable time in solitude. Older people often have strong attachments to their homes (e.g., Wiles et al., 2009), and remaining in their homes can be strongly associated with feelings of independence, which is seen as extremely valuable (Haak, Fänge, Iwarsson, & Dahlin-Ivanoff, 2007b).

Gillsjö, Schwartz-Barcott, and von Post (2011) stress that home is the place that the oldest old adult cannot imagine living without, but might be forced to leave. The same fear was present in our study, as the women might be forced to leave home because of decreasing health, and especially with the development of dementia. Remaining in their own homes gives rise to *feeling secure* for the oldest old women. Dahlin-Ivanoff et al. (2007) found that home means security and freedom for oldest old persons living alone. Home is considered extremely valuable for the oldest old women in the present study for major reasons. They have taken root in the houses and surrounding area, because these have been their homes and workplaces since early adulthood, and this is where they have raised their children.

Fänge and Dahlin-Ivanoff (2009) found that home can be the hub of health in old age, even though one's existing life situation could rapidly change. This is congruent with the findings of our study. Oldest old women felt better and felt there would always be a solution when they got home, even though they knew their life situation could quickly change. Home can, in the aging process and in widowhood, become invested with new meanings and functions and become an emotional center of older women's lives (Cristoforetti, Gennai, & Rodeschini, 2011). It can mean security and freedom for single oldest old persons (Dahlin-Ivanoff et al., 2007). Long-term emotional attachments to environmental surroundings can contribute to well-being in old age (Taylor, 2001), and the oldest old women in our study were strongly attached to their homes.

One coping strategy for oldest old rural persons to remain at home in old age is to change the environment and adapt everyday practices and patterns so autonomy can be maximized (Hinck, 2004). This was present for the oldest old women in our study, as they adapted despite declining health and new impairments (e.g., by using various aids). Faith in care and treatment is one factor that gives rise to feelings of security among women with chronic heart failure (Burström, Brännström, Boman, & Strandberg, 2011). Patients feel secure when formal caregivers are punctual and skilled, but on the other hand insecure when they received less help than needed (Efraimsson, Höglund, & Sandman, 2001). In our study, the oldest old women felt secure because of received care when necessary and being able to call as needed. No feelings of insecurity toward home nursing care were revealed.

The oldest old women in our study appreciated time alone in solitude. Solitude is an experience of disengagement from other people (Koch, 1997), or, as Gotesky defined it, “Solitude is the state or condition of living alone, in any of its many forms, without the pain of loneliness or isolation being an intrinsic component of the state or condition” (Gotesky, 1965, p. 236). Solitude is a primary mode of human experience, and the virtues of solitude are “freedom, attunement to self, attunement to nature, and creativity” (Koch, 1997, p. 99).

Even if the oldest old women in our study did not become alone by choice, they remained alone by choice. Most women had been alone for many years, some since their forties. By being able to treasure and find meaning in their aloneness, they were able to find new ways of connecting. They connected to themselves by finding new meaning in their lives as the oldest old women in rural areas. They connected (e.g., to nature and memories) and found new meanings through solitude. Or, as Frankl (1971) emphasizes, they created a new world by connecting the past and the future and imbuing it with new meaning. Our study implies that the oldest old women in rural areas manage this by using their inner strength to see the positive in their lives and come to terms with their losses. Achieving a full life in solitude implies having the right thoughts, and by that Powys (1974) means the thoughts that give you a calm happiness. The women in our study faced life's adversities, but they still managed to see the positive aspects of aging and loss. They were satisfied with their lives, and primarily lived in solitude in their homes. By retiring to solitude, one can manage to be aware of and affect one's thoughts (Powys, 1974), as well as be aware of new meanings in life so one can live more fully (Moustakas, 1961), as the women in our study did.

Home is a place where older rural women wish to be and where they pursue health through physical, creative, and intellectual endeavors as well as solitude (Hayes, 2006). Hinck (2004) emphasizes that home for the oldest old rural adults was a physical space and a state of being allowed to be self-determinant; it allowed them to remain connected to their past. They, in fact, treasured the solitude of living alone. Older women living at home alone in rural areas want to balance their need for connection with others with their desire for solitude and privacy, and the desire to continue to live at home (Letvak, 1997). This was also the case in the present study, even if they received care from home nursing care and were homebound because of declining health and impairments.

In order to be satisfied in life as an oldest old woman living alone in rural areas, our study implies that one advantage is the ability to appreciate aloneness as solitude, and to use time in solitude to find new meaning in life. By using their inner strength to face losses and declining health, the oldest old women managed to create a full life with meaning in solitude, as they were able to “bounce back” in the face of adversities.

### Methodological considerations

When using phenomenological hermeneutics, the aim is to reveal the essential meaning of “being in the world” (Lindseth & Nordberg, 2004) (i.e., we have to get access to the person's lifeworld). The gateway to a person's lifeworld runs through the narrative, with its symbols and metaphors, and has to be interpreted by using explanation and understanding (Ricoeur, 1976). In the hermeneutic tradition, issues of trustworthiness are a matter of difficulty throughout the interpretations—not at finding the truth. So where the first naïve understanding can be seen as a guess (Ricoeur, 1976), the structural analyses can be seen as both a validation of our guess (Lindseth & Nordberg, 2004) and an attempt to explain the present text. This means that our interpretation is one of several possible interpretations, and cannot be seen as the absolute truth, because a text can be interpreted in different ways (Ricoeur, 1976).

In the phenomenological hermeneutic methodological stages, there is an ongoing movement between the text as a whole and parts (Lindseth & Nordberg, 2004). The first author carried out the initial analysis; however, all three authors have reflected on and continuously and critically worked with the assessments until a consensus was reached. Our interpretation represents what we have found to be the most useful way of understanding this phenomenon.

Eleven oldest old women participated in the present study, and it could be argued that's an insufficient number of participants for reaching a meaning of being an oldest old woman living alone in rural areas and receiving home nursing care. However, in lifeworld research, the complexity of the phenomena is more crucial than the number of participants (Dahlberg, Dahlberg, & Nyström, 2008). In the present study, the participants’ occupational background, municipalities, and amount of home nursing care received varied.

Not all requested oldest old women wanted to participate in the study, and one can think that only the most positive, satisfied, and resilient women were willing to participate. Some significant voices have likely been lost because of fears such as that of home nursing care finding out about negative comments. However, this study offers one perspective on understanding the phenomenon of living alone as an oldest old woman and receiving home nursing care in rural areas.

### Conclusion and implications for nursing practice

Even if oldest old people living alone in rural areas are considered a vulnerable population, nurses need to be aware that this population varies tremendously. The women in our study, for example, showed strength and the ability to have a perceived good life in solitude. Oldest old persons living alone can experience loneliness, but that is not the whole picture, as the present study shows that several oldest old women are satisfied with their lives in aloneness in rural areas. This means that nurses need to approach them on an individual basis, and also be aware of their own importance as a social contact, as they may be the only one visiting several oldest old homebound women on a regular basis.

Despite the limited number of participants in the present study, it revealed that oldest old women living alone in rural areas have several resources, such as, for example, inner strength. By recognizing and supporting their recourses, the home nurse can contribute to oldest old women remaining longer in their beloved homes.

## References

[CIT0001] Alex L (2010). Resilience among very old men and women. Journal of Research in Nursing.

[CIT0002] Andersson M, Hallberg I. R, Edberg A-K (2008). Old people receiving municipal care, their experiences of what constitutes a good life in the last phase of life: A qualitative study. International Journal of Nursing Studies.

[CIT0003] Andreassen K. K, Mørk E (2011). Befolkningens størrelse og aldersfordeling i. Seniorer i Norge 2010. Statistisk sentralbyrå. Statistics Norway.

[CIT0004] Berg N. G, Lysgård H. K (2004). Ruralitet og urbanitet – bygd og by. Plan: *Tidsskrift for samfunnsplanlegging*.

[CIT0005] Borglin G, Edberg A-K, Hallberg I. R (2005). The experience of quality of life among older people. Journal of Aging Studies.

[CIT0006] Burström M, Brännström M, Boman K, Strandberg G (2011). Life experiences of security and insecurity among women with chronic heart failure. Journal of Advanced Nursing.

[CIT0007] Chandler J, Williams M, Maconachie M, Collett T, Dodgeon B (2004). Living alone: Its place in household formation and change. Sociological Research Online.

[CIT0008] Creswell J. W (2009). Research design. Qualitative, quantitative, and mixed methods approaches.

[CIT0009] Cristoforetti A, Gennai F, Rodeschini G (2011). Home sweet home: The emotional construction of places. Journal of Aging Studies.

[CIT0010] Dahlberg K, Dahlberg H, Nyström H (2008). Reflective lifeworld research.

[CIT0011] Dahlin-Ivanoff S, Haak M, Fänge A, Iwarsson S (2007). The multiple meaning of home as experienced by very old Swedish people. Scandinavian Journal of Occupational Therapy.

[CIT0012] Dale B, Sævareide H. I, Kirkevold M, Söderhamn O (2011). Older home-living patients’ perceptions of received home nursing and family care. Nordisk sygeplejeforskning.

[CIT0013] Dale B, Söderhamn U, Söderhamn O (2012). Life situation and identity among single older home-living people: A phenomenological- hermeneutic study. International Journal of Qualitative Studies of Health and Well-Being.

[CIT0014] de Jonge D. M, Jones A, Phillips R, Chung M (2011). Understanding the essence of home: Older people's experience of home in Australia. Occupational Therapy International.

[CIT0015] Efraimsson E, Höglund I, Sandman P. O (2001). The everlasting trial of strength and patience’: Transitions in home care nursing as narrated by patients and family members. Journal of Clinical Nursing.

[CIT0016] Fagerström L (2010). Positive life orientation-an inner health resource among older people. Scandinavian Journal of Caring Sciences.

[CIT0017] Fänge A, Dahlin-Ivanoff S (2009). The home is the hub of health in very old age: Findings from the ENABLE-AGE Project. Archives of Gerontology and Geriatrics.

[CIT0018] Frankl V (1971). The will to meaning.

[CIT0019] Gattuso S (1996). The meaning of home for older women in rural Australia. Australian Journal of Ageing.

[CIT0020] Gaymu J, Springer S (2010). Living condition and life satisfaction of older European living alone: A gender and cross-country analysis. Aging and Society.

[CIT0021] Gillsjö C, Schwartz-Barcott D, von Post I (2011). Home: The place the older adult can not imagine living without. BMC Geriatrics.

[CIT0022] Gotesky R, Edie J (1965). Aloneness, loneliness, solitude. An invitation to phenomenology.

[CIT0023] Haak M, Dahlin-Ivanoff S, Fänge A, Sixsmith J, Iwarsson S (2007a). Home as the locus and origin for participation: Experiences among very old Swedish people. Occupation, Participation and Health.

[CIT0024] Haak M, Fänge A, Iwarsson S, Dahlin-Ivanoff S (2007b). Home as signification of independence and autonomy: Experiences among very old Swedish people. Scandinavian Journal of Occupational Therapy.

[CIT0025] Hayes P. A (2006). Home is where their health is: Rethinking perspectives of informal and formal care by older rural Appalachian woman who live alone. Qualitative Health Research.

[CIT0026] Howell S. C, Altman I, Churchman A (1994). Environment and the aging woman: Domains of choice. Women and the environment.

[CIT0027] Hinck S (2004). The lived experience of oldest-old rural adults. Qualitative Health Research.

[CIT0028] Hinck S (2007). The meaning of time in oldest-old age. Holistic Nursing Practise.

[CIT0029] Johansen E, Fagerström L (2010). An investigation of the role nurses play in Norwegian home care. British Journal of Community Nursing.

[CIT0030] Johnson M. L, Bengtson V. L, Coleman P. G, Kirkwood T. B. L (2005). The Cambridge handbook of age and ageing.

[CIT0031] Koch L (1997). Solitude, a philosophical encounter.

[CIT0032] Larsson Å, Haglund L, Hagberg J-E (2009). Doing everyday life-experiences of the oldest old. Scandinavian Journal of Occupational Therapy.

[CIT0033] Leith K. H (2006). ‘Home is where the heart is … or is it?’ A phenomenological exploration of the meaning of home for older women in congregate housing. Journal of Aging Studies.

[CIT0034] Letvak S (1997). Relational experiences of elderly women living alone in rural communities: A phenomenologic inquiry. The Journal of the New York State NursesAssociation.

[CIT0035] Lindseth A, Norberg A (2004). A phenomenological hermeneutical method for researching lived experience. Scandinavian Journal of Caring Sciences.

[CIT0036] Mallett S (2004). Understanding home: A critical review of the literature. The Sociological Review.

[CIT0037] Mishler G (1986). Research interviewing: Context and narrative.

[CIT0038] Moe A, Ekker K, Enmarker I (2013a). A description of resilience for Norwegian home-living chronically ill oldest older persons. Open Journal of Nursing.

[CIT0039] Moe A, Hellzen O, Enmarker I (2013b). The meaning of receiving help from home nursing care. Nursing Ethics.

[CIT0040] Moustakas C. E (1961). Loneliness.

[CIT0041] Ness T. M, Hellzen O, Enmarker I (2014). ‘Struggling for independence’: The meaning of being an oldest old man in a rural area. Interpretation of oldest old men's narrations. International Journal of Qualitative Studies of Health and Well-Being.

[CIT0042] Oswald F, Wahl H. W, Rowles G. D, Chaudhury H (2005). Dimensions of the meaning of home in later life. Home and identity in late life.

[CIT0043] Otnes B, Mørk E (2011). Helse i. Seniorer i Norge 2010. Statistisk sentralbyrå. Statistics Norway.

[CIT0044] Patton M. Q (2002). Qualitative research & evaluation methods.

[CIT0045] Powys J. C (1974). A philosophy of Solitude.

[CIT0046] Ricoeur P (1976). Interpretation theory, discourse and surplus of meaning.

[CIT0047] Russell C (2007). What do older women and men want?: Gender differences in the “Lived experience” of aging. Current Sociology.

[CIT0048] Sandelowski M (1994). We are the stories we tell: Narrative knowing in nursing practice. Journal of Holistic Nursing.

[CIT0049] Shenk D, Kuwahara K, Zablotsky D (2004). Older women's attachment to their home and possessions. Journal of Aging Studies.

[CIT0050] Silfverberg G, Ternestedt B-M, Silfverberg G (2007). Hemmet och vårdetiken. Hemmets vårdetik, Om vård av äldre I livets slutskede.

[CIT0051] Sundin K, Bruce E, Barremo A. S (2010). Elderly women's experiences of support when living with congestive heart failure. International Journal of Qualitative Studies in Health Well-Being.

[CIT0052] Söderhamn U, Dale B, Söderhamn O (2011). Narrated lived experiences of self-care and health among rural-living older persons with a strong sense of coherence. Psychology Research and Behaviour Management.

[CIT0053] Tarricone R, Tsours A. D (2008). The solid facts: Home care in Europa.

[CIT0054] Taylor S. A. P (2001). Place identification and positive realities of aging. Journal of Cross-Cultural Gerontology.

[CIT0055] Windle G (2011). What is resilience? A review and concept analysis. Reviews in Clinical Gerontology.

[CIT0056] Wiles J. L, Allen R. E. S, Palmer A. J, Hayman K. J, Keeling S, Kerse N (2009). Older people and their social spaces: A study of well-being and attachment to place in Aotearoa New Zealand. Social Science & Medicine.

[CIT0057] World Medical Association (2008). WMA declaration of Helsinki – Ethical principles for medical research involving human subjects.

